# Distinct Immune Responses Elicited From Cervicovaginal Epithelial Cells by Lactic Acid and Short Chain Fatty Acids Associated With Optimal and Non-optimal Vaginal Microbiota

**DOI:** 10.3389/fcimb.2019.00446

**Published:** 2020-01-10

**Authors:** David J. Delgado-Diaz, David Tyssen, Joshua A. Hayward, Raffi Gugasyan, Anna C. Hearps, Gilda Tachedjian

**Affiliations:** ^1^Disease Elimination Program and Life Sciences Discipline, Burnet Institute, Melbourne, VIC, Australia; ^2^Department of Microbiology, Monash University, Clayton, VIC, Australia; ^3^Department of Immunology, Monash University, Melbourne, VIC, Australia; ^4^Department of Infectious Diseases, Monash University, Melbourne, VIC, Australia; ^5^Department of Microbiology and Immunology at the Peter Doherty Institute for Infection and Immunity, University of Melbourne, Melbourne, VIC, Australia

**Keywords:** vaginal microbiota metabolites, lactic acid, bacterial vaginosis, inflammation, short chain fatty acids, succinic acid, cervicovaginal epithelium

## Abstract

Non-optimal vaginal microbiota, as observed in bacterial vaginosis (BV), is typically characterized by a depletion of beneficial lactobacilli and an abundance of numerous anaerobes. These non-optimal conditions are associated with subclinical cervicovaginal inflammation and an increased risk of HIV infection compared to women colonized with optimal vaginal microbiota dominated by lactobacilli. Lactic acid (LA) is a major organic acid metabolite produced by vaginal lactobacilli that elicits anti-inflammatory effects from cervicovaginal epithelial cells and is dramatically depleted during BV. However, it is unclear if LA retains its anti-inflammatory activity in the presence of vaginal microbiota metabolites comprising short chain fatty acids (SCFAs) and succinic acid, which are also produced by an optimal vaginal microbiota. Furthermore, the immunomodulatory effect of SCFAs and succinic acid on cervicovaginal epithelial cells at higher concentrations present during BV is unknown. Here we report that in the presence of physiologically relevant concentrations of SCFAs and succinic acid at pH 3.9 (as found in women with lactobacillus-dominated microbiota) LA induced an anti-inflammatory state in cervicovaginal epithelial cells and inhibited inflammation elicited by the toll-like receptor (TLR) agonists polyinosinic:polycytidylic acid and Pam3CSK4. When cervicovaginal epithelial cells were treated with a vaginal microbiota metabolite mixture representative of BV, containing a lower concentration of LA but higher concentrations of SCFA/succinic acid at pH 7, no anti-inflammatory was observed. Rather, the vaginal microbiota metabolite mixture representative of BV dysregulated the immune response of cervicovaginal epithelial cells during prolonged and sustained treatments. This was evidenced by increased basal and TLR-induced production of pro-inflammatory cytokines including tumor necrosis factor-α, but decreased basal production of chemokines including RANTES and IP-10. Further characterization of individual components of the BV vaginal microbiota mixture suggested that acetic acid is an important vaginal microbiota metabolite capable of eliciting diverse immunomodulatory effects on a range of cervicovaginal epithelial cell targets. These findings indicate that elevated levels of SCFAs are a potential source of cervicovaginal inflammation in women experiencing BV, and support the unique anti-inflammatory properties of LA on cervicovaginal epithelial cells as well as a role for LA or LA-producing lactobacilli to reverse genital inflammation associated with increased HIV risk.

## Introduction

The lower female reproductive tract epithelium plays important roles as a physical and immunological barrier against infection (Anderson et al., 2014); however its function is influenced by many factors including the effects of commensal vaginal microbiota and their metabolic products (O'Hanlon et al., 2011, 2013; Ravel et al., 2011; Aldunate et al., 2013; Anderson et al., 2014; Hearps et al., 2017; Tachedjian et al., 2017). The lower female reproductive tract microbiota is dynamic (Gajer et al., 2012) and can vary considerably between women of different ethnicities and geographical locations (Ravel et al., 2011; Gajer et al., 2012; Gosmann et al., 2017), although a predominance of beneficial lactobacilli is considered an optimal microbiota that is associated with eubiosis and favorable reproductive and sexual health outcomes (Aldunate et al., 2015; Fettweis et al., 2019; McKinnon et al., 2019).

Lactic acid (LA) is an organic acid metabolite predominately produced by lactobacilli (Boskey et al., 2001) and is present in the lower female reproductive tract at a concentration of ~110 mM (~1.0% ± 0.2% w/v) and a pH < 4.5 in women with lactobacillus-dominated microbiota (Boskey et al., 2001; O'Hanlon et al., 2013). There are two isoforms of LA, L-LA, and D-LA, which are produced in different ratios within the lower female reproductive tract by various *Lactobacillus* spp. (Witkin et al., 2013). At a vaginal pH below its pK_a_ (3.86), LA is predominantly present in its protonated form (Aldunate et al., 2013) which elicits an anti-inflammatory response from cervicovaginal epithelial cells *in vitro* (Hearps et al., 2017). LA has antimicrobial activities against human immunodeficiency virus (HIV) (Aldunate et al., 2013), herpes simplex virus (Conti et al., 2009; Isaacs and Xu, 2013), *Neisseria gonorrhoeae* (Graver and Wade, 2011), *Chlamydia trachomatis* (Gong et al., 2014), and bacteria associated with bacterial vaginosis (BV) (O'Hanlon et al., 2011), and inhibits the infection of epithelial cells with *Chlamydia trachomatis in vitro* (Edwards et al., 2019).

In contrast, the lower female reproductive tract of women colonized with a dysbiotic or non-optimal microbiota, such as in BV, is dominated by anaerobes, a dramatic depletion of beneficial lactobacilli and an elevated vaginal pH > 4.5. This altered microbiota is associated with distinct vaginal concentrations of organic acid metabolites including short chain fatty acids (SCFA) and succinic acid produced by commensal bacteria (reviewed in Aldunate et al., 2015). In addition to LA, which is the predominant metabolite of a lactobacillus-dominated microbiota, acetic and succinic acid, and to a lesser extent propionic and butyric acid, are major microbiota metabolites found within the lower female reproductive tract, particularly during BV where their concentration increases dramatically, up to ~120 and 20 mM for acetic and succinic acid, respectively (Al-Mushrif et al., 2000; Chaudry et al., 2004; Mirmonsef et al., 2011, 2012b; Gajer et al., 2012).

BV is also associated with subclinical genital inflammation (Eschenbach et al., 1988) and an increased risk of transmitting and contracting HIV (Eschenbach et al., 1988; Atashili et al., 2008; Cohen et al., 2012). Women diagnosed with Nugent-BV, as determined by the Nugent score that enumerates bacterial morphotypes using a Gram stain (McKinnon et al., 2019), are at a 1.53-fold (95% CI = 1.24–1.89) higher risk of acquiring HIV than women without BV (Low et al., 2011), while young South African women colonized with a pro-inflammatory, diverse vaginal microbiota, as determined by next generation sequencing i.e., Molecular-BV (McKinnon et al., 2019), are at a 4.4-fold (95% CI = 1.17–16.61) higher risk of acquiring HIV than women colonized by *L. crispatus*-dominated microbiota (Gosmann et al., 2017). Another study in African women showed that bacterial diversity and specific BV-associated taxa (e.g., *Eggerthella* species type I, *Parvimonas* species types I and 2 and *Megasphaera* spp.) were associated with increased risk of HIV acquisition (McClelland et al., 2018). The pro-inflammatory female reproductive tract milieu associated with non-optimal vaginal microbiota is thought to increase HIV susceptibility by recruiting activated HIV target cells to the genital mucosa, decreasing epithelial integrity and changing the barrier properties of cervicovaginal mucus (Lewis et al., 2013; Borgdorff et al., 2016; Zevin et al., 2016; Gosmann et al., 2017).

Although the mechanisms contributing to the pro-inflammatory cervicovaginal milieu due to non-optimal vaginal microbiota (e.g., BV) have not been completely elucidated, overgrowth of vaginal commensal anaerobic bacteria such as *Gardenerella vaginalis, Prevotella* spp*., Atopobium vaginae*, and *Molibuncus* spp. may expose vaginal epithelial cells to pathogen-associated molecular patterns, stimulating pathogen recognition receptors such as toll-like receptors (TLRs) and eliciting an inflammatory response (Lavelle et al., 2010; Byrne et al., 2015; Gosmann et al., 2017). Organic acid metabolic products from BV-associated bacteria such as SCFAs and succinic acid at concentrations observed during BV (Al-Mushrif et al., 2000; Chaudry et al., 2004; Mirmonsef et al., 2011) elicit a pro-inflammatory response from peripheral blood cells and enhance TLR-associated inflammation (Mirmonsef et al., 2012a); however the impact of SCFAs on cervicovaginal epithelial cells has not been reported.

We have previously shown that protonated LA at low pH (3.9), present during vaginal eubiosis (O'Hanlon et al., 2013; Tyssen et al., 2018), elicits an anti-inflammatory response and dampens TLR-induced pro-inflammatory responses from cervicovaginal epithelial cells (Hearps et al., 2017). However, it is unknown whether other vaginal microbial metabolic products present in the lower female reproductive tract such as SCFAs and succinic acid modulate this effect, or if they have immunomodulatory effects of their own. Accordingly, we investigated whether L-LA in combination with other vaginal microbiota metabolites, and at a pH relevant to vaginal eubiosis, maintains its anti-inflammatory effects. In addition, we investigated if individual or combinations of SCFAs and succinic acid, at conditions typically observed in BV, modulate the production of cytokines and chemokines relevant to increased HIV risk from cervicovaginal epithelial cells.

## Materials and Methods

### Cervicovaginal Epithelial Cell Culture and Stimulations

Ectocervical Ect1/E6E7 (Ect) and vaginal VK2/E6E7 (VK2) epithelial cells were obtained from the American Type Culture Collection (Manassas, VA) and maintained in serum-free keratinocyte medium, supplemented with 0.1 ng /ml human recombinant epithelial growth factor, 0.05 mg/ml bovine pituitary extract (all from Life Technologies, Carlsbad, CA), 0.4 mM CaCl_2_, 50 U/ml penicillin and 50 μg/ml streptomycin. Primary human cervicovaginal epithelial cells derived from discarded surgical tissue (Ayehunie et al., 2006) were obtained from MatTek (Ashland, MA) and were maintained in media as above but with 10 ng/ml human recombinant epithelial growth factor.

Cervicovaginal epithelial cells (100,000 per well) were seeded into either 6.5 mm diameter polycarbonate transwell inserts containing 0.4 μm pores (Corning, Corning, NY) in 24-well tissue culture plates or into 96-well tissue culture plates and cultured for 7 days to obtain a confluent cell monolayer. Epithelial monolayers were treated with combinations of vaginal microbiota metabolites, comprising L-LA, butyric acid, succinic acid (all from Sigma Aldrich, St. Louis, MO), acetic acid (Merck, Kenilworth, NJ), and propionic acid (MP Biomedicals, Santa Ana, CA). To mimic a lactobacillus-dominant microbiota (defined as eubiosis), cells were treated with media containing the predominate metabolites found during eubiosis and at concentrations and pH representative of this state. We assessed L-LA at 33 mM given our previous work showing that this concentration had significant immunomodulatory effects on cervicovaginal cells but minimal impacts on cell viability (Hearps et al., 2017). Acetic, succinic, propionic, and butyric acids are typically present at low to undetectable levels in vaginal fluid from women with eubiosis (Al-Mushrif et al., 2000; Mirmonsef et al., 2011; O'Hanlon et al., 2013) but can reach levels up to ~4.6 mM for acetic acid (O'Hanlon et al., 2013) and 0.6 mM for succinic acid (Al-Mushrif et al., 2000) in women with lactobacillus-dominated vaginal microbiota, thus concentrations of 4 mM for acetic and 1 mM for succinic, butyric and propionic acid were assessed as part of the eubiosis mixture. Eubiosis treatment media was adjusted to pH 3.9 to mimic the physiological pH of women with lactobacillus-dominated microbiota (O'Hanlon et al., 2013). During BV, vaginal concentrations of acetic, succinic, propionic and butyric acid, as well as vaginal pH, are substantially increased whilst LA levels are decreased (Spiegel et al., 1983; Al-Mushrif et al., 2000; Mirmonsef et al., 2011; Gajer et al., 2012). To mimic these conditions, cells were treated with media containing L-LA 6 mM (reduced to ~20% of that in the eubiosis mixture to reflect the approximate 5-fold reduction in LA concentration observed in women with BV), acetic acid 100 mM, succinic acid 20 mM, propionic acid 4 mM, and butyric acid 4 mM at pH 7. These concentrations of SCFAs and succinic acid were selected to reflect those reported in women experiencing BV, where levels of acetic acid can reach up to ~120 mM (Chaudry et al., 2004; Mirmonsef et al., 2011), succinic acid up to ~22 mM (Al-Mushrif et al., 2000) and propionic and butyric acids up to ~4 mM (Al-Mushrif et al., 2000). This BV metabolite mixture was adjusted to pH 7 before adding to cells, to reflect the upper limit of vaginal pH commonly found in women with BV (Brabin et al., 2005; Sánchez-Hernández et al., 2012). To assess the effect of individual vaginal microbiota metabolites in the context of BV, cells were treated with each metabolite alone at the same concentration as in the BV mix. Vaginal microbiota metabolites were added in the absence or presence of the TLR1/2 agonist Pam3CSK4 (Pam, 1 μg/ml) or the TLR3 agonist high-molecular weight polyinosinic:polycytidylic (PIC, 20 μg/ml) (both from InvivoGen, San Diego, CA) to simulate pathogen challenge. The pH stated for each treatment media reflects that of the media added to the apical chamber for experiments conducted in transwells, and of the entire well for experiments conducted in culture plates.

Cells in transwell inserts were stimulated apically with vaginal microbiota metabolite-containing media for 1 h, washed with PBS, fresh media (containing no stimulant) was then added and cells were incubated for a further 18 h. Previous optimization identified the pH of treatment media in the apical chamber was maintained during this 1 h stimulation period (Hearps et al., 2017). For experiments involving prolonged stimulation with BV-relevant SCFA, transwells were not able to maintain the desired conditions for these extended periods due to diffusion between apical and basal compartments, thus cells were treated in tissue culture plates. For these experiments, cells cultured in 96-well plates were stimulated continuously with treatment media for 24 h. Supernatants were collected from the wells or the apical chamber of the transwell inserts and stored at −80°C for subsequent cytokine analysis. Cell viability was assessed following stimulation and supernatant collection using the CellTiter 96 Aqueous One Solution Cell proliferation assay (Promega, Madison, WI) as published (Hearps et al., 2017). Cell viability for all treatments remained above 70% of untreated cells (data not shown and as previously reported for LA) (Hearps et al., 2017) indicating minimal effects on cell viability under the conditions tested.

### Cytokine and Chemokine Quantitation

The production of cytokines interleukin (IL)-1 receptor antagonist (IL-1RA), IL-6, tumor necrosis factor α (TNFα), IL-1β, and chemokines IL-8, C-C motif chemokine ligand 5 (RANTES), interferon gamma-induced protein 10 (IP-10), and macrophage inflammatory protein 3 alpha (MIP-3α) in culture supernatant was quantified using ProcartaPlex Multiplex Immunoassays (Affymetrix, Santa Clara, CA). Samples were analyzed on a Luminex 200 (Bio-Rad, Hercules, CA), or MAGPIX system (Merck Millipore, Burlington, MA), and analysis was performed using manufacturer-provided software. For some treatments, values for IP-10 were above the limit of quantification of the assay and were assigned the maximum value of the standard curve.

## Results

### The Anti-inflammatory Effect of L-LA on Cervicovaginal Epithelial Cells Persists in the Presence of Other Organic Acid Metabolites Relevant to Vaginal Eubiosis

Our previous studies examined the immunomodulatory effect of LA alone on cervicovaginal epithelial cells (Hearps et al., 2017); however LA exists in combination with other organic acid metabolites from female reproductive tract-resident bacteria in the vaginal lumen (Aldunate et al., 2015). Our previous study demonstrated that L- and D-LA elicit similar responses from cervicovaginal epithelial cells with regard to cytokines and chemokines associated with increased risk of HIV transmission in women (Masson et al., 2015; Hearps et al., 2017). Accordingly, we performed experiments using one of the LA isomers (L-LA). To determine if L-LA maintains its anti-inflammatory effect when combined with other organic acid metabolites, cervicovaginal epithelial cells were treated in transwell inserts with a combination of vaginal microbiota metabolites relevant to eubiosis with and without TLR stimulation for 1 h, shown previously to be sufficient for L-LA to elicit its anti-inflammatory effects (Hearps et al., 2017), and cytokine and chemokine production were analyzed 18 h post-treatment. The eubiotic condition tested represents the maximum level of SCFAs and succinic acid measured in women carrying an optimal vaginal microbiota (Al-Mushrif et al., 2000; Mirmonsef et al., 2011, 2012b; O'Hanlon et al., 2013). Prior to addition to the apical compartment, the pH of treatment media was adjusted to 3.9 to mimic the physiological acidity in women with an optimal, lactobacillus-dominated vaginal microbiota (Aldunate et al., 2013).

**Figure 1:**
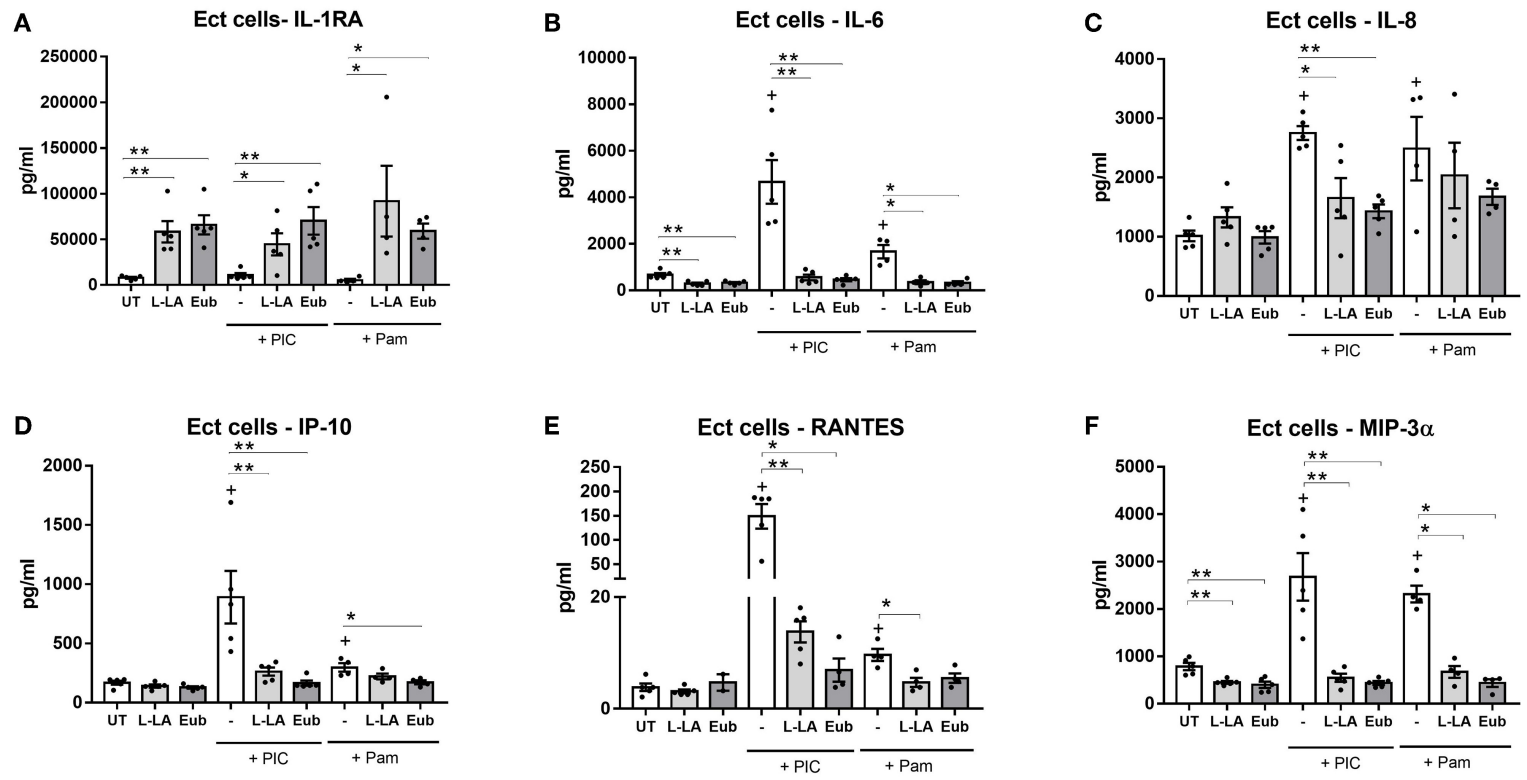
Eubiotic vaginal microbiota metabolite mixture containing L-LA elicits a similar anti-inflammatory effect on Ect cells compared to L-LA alone. Ect cells seeded in transwells were apically treated with a mixture of vaginal microbiota metabolites at pH 3.9 recapitulating vaginal eubiosis (Eub) and L-LA alone in the absence or presence of the TLR agonists PIC (TLR3) and Pam (TLR1/2) as indicated. Cells were stimulated for 1 h and supernatant collected from the apical compartment after an additional 18 h of culture. Production of IL-1RA **(A)**, IL-6 **(B)**, IL-8 **(C)**, IP-10 **(D)**, RANTES **(E)**, and MIP-3α **(F)** was quantified using a luminex multiplex assay. Graphs show mean and standard error of the mean from *n* = 4 independent assays. Mann-Whitney tests were used to estimate statistical significance between treatment groups. + denotes a significant difference (*p* < 0.05) as compared to untreated (UT) cells. *P* ≤ 0.05 and ≤ 0.01 are denoted as * and **, respectively. L-LA, L-isomer of lactic acid; TLR, toll-like receptor; PIC, polyinosinic:polycytidylic acid; Pam, Pam3CSK4.

Ectocervical (Ect) and primary ectocervical cells, treated with L-LA in combination with the eubiotic vaginal microbiota metabolite mixture elicited an 8.5-fold (*p* = 0.01) and 2.6-fold (*p* = 0.02) increase in the production of the anti-inflammatory cytokine IL-1RA as compared to untreated Ect and primary cells, respectively, which was similar to the effect elicited by L-LA alone (Figure 1A and Supplementary Figure 1A). A heightened production of IL-1RA was also observed from vaginal VK2 cells treated with eubiotic vaginal microbiota metabolite mixture containing L-LA compared to untreated cells, although this did not reach statistical significance (Supplementary Figure 2A). Furthermore, the production of IL-1RA by eubiotic and L-LA treatments was also observed in the presence of TLR1/2 (Pam) and TLR3 (PIC) agonists, which mimic bacterial and viral challenge, respectively (Figure 1A and Supplementary Figures 1A, 2A).

Basal production of IL-6 and MIP-3α from Ect cells was significantly decreased by both the eubiotic treatment and L-LA (*p* = 0.01 for both, Figures 1B,F) consistent with our previous findings (Hearps et al., 2017), and a similar trend for IL-6 was also seen in VK2 cells (Supplementary Figure 2B). Similar to L-LA alone, the eubiotic treatment elicited a minimal 2-fold increase in the production of IL-1β from all cell types (Supplementary Figures 1F, 2F, 3A), which physiologically is likely to be mitigated by the substantially higher production of the antagonist IL-1RA elicited by L-LA (7420-, 5812-, and 10166- fold excess of IL-1RA to IL-1β in Ect, VK2 and primary cells, respectively (Figure 1 and Supplementary Figures 1, 2). Taken together, these data indicate that the anti-inflammatory effect of L-LA on unstimulated cervicovaginal epithelial cells persists within a complex mixture of vaginal microbiota organic acids representing a eubiotic vaginal environment.

### A Eubiotic Vaginal Microbiota Metabolite Mixture Containing L-LA Inhibits Pro-inflammatory Responses From TLR Stimulated Cervicovaginal Epithelial Cells

Genital inflammation, driven by a highly diverse vaginal microbiota, and increased cervicovaginal levels of pro-inflammatory cytokines and chemokines are associated with higher levels of HIV transmission (Masson et al., 2015; Gosmann et al., 2017). Beneficial vaginal lactobacilli (e.g., *L. crispatus*) dampen inflammatory and inhibit pro-inflammatory responses mediated by TLR agonists (Rose et al., 2012; Doerflinger et al., 2014) and vaginal pathogens (Santos et al., 2018), and we have demonstrated that this effect is mediated, at least in part, by LA (Hearps et al., 2017). Here, we asked whether this effect is maintained in the presence of physiological concentrations of other vaginal organic acid metabolites.

Apical stimulation of Ect cells with PIC and Pam, elicited an increased production of pro-inflammatory immune mediators including IL-6, IL-8, IP-10, RANTES, and MIP-3α, with PIC eliciting a greater response than Pam stimulation except for IL-8 and MIP-3α (Figures 1B–F). Similar to the effect of L-LA alone, in the context of PIC stimulation the eubiotic treatment significantly dampened the production of IL-6, IL-8, IP-10, MIP-3α (*p* < 0.01 for all) and RANTES (*p* = 0.02) from Ect cells (Figures 1B–F). A similar inhibitory effect was observed for Pam stimulated Ect cells, which was statistically significant for IL-6, IP-10, and MIP-3α production (*p* = 0.03 for all). TNFα production from Ect cells was largely below the limit of quantification, except for PIC-stimulated cells, with this response being completely inhibited by L-LA (Supplementary Figure 3B).

We next confirmed this result in VK2 and primary cervicovaginal cells and observed a similar ability of the eubiotic treatment to inhibit TLR-mediated inflammation, similar to that observed with L-LA alone (Supplementary Figures 1, 2). These data demonstrate that the ability of L-LA to mitigate the pro-inflammatory response of cervicovaginal epithelial cells to TLR agonists mimicking bacterial and viral PAMPs is retained in the presence of physiological concentrations of vaginal microbiota organic acid metabolites found in women with a lactobacillus-dominated microbiota.

### Short Exposure of Cervicovaginal Epithelial Cells to Vaginal Microbiota Metabolites Associated With BV Do Not Elicit Immunomodulatory Effects

**Figure 2:**
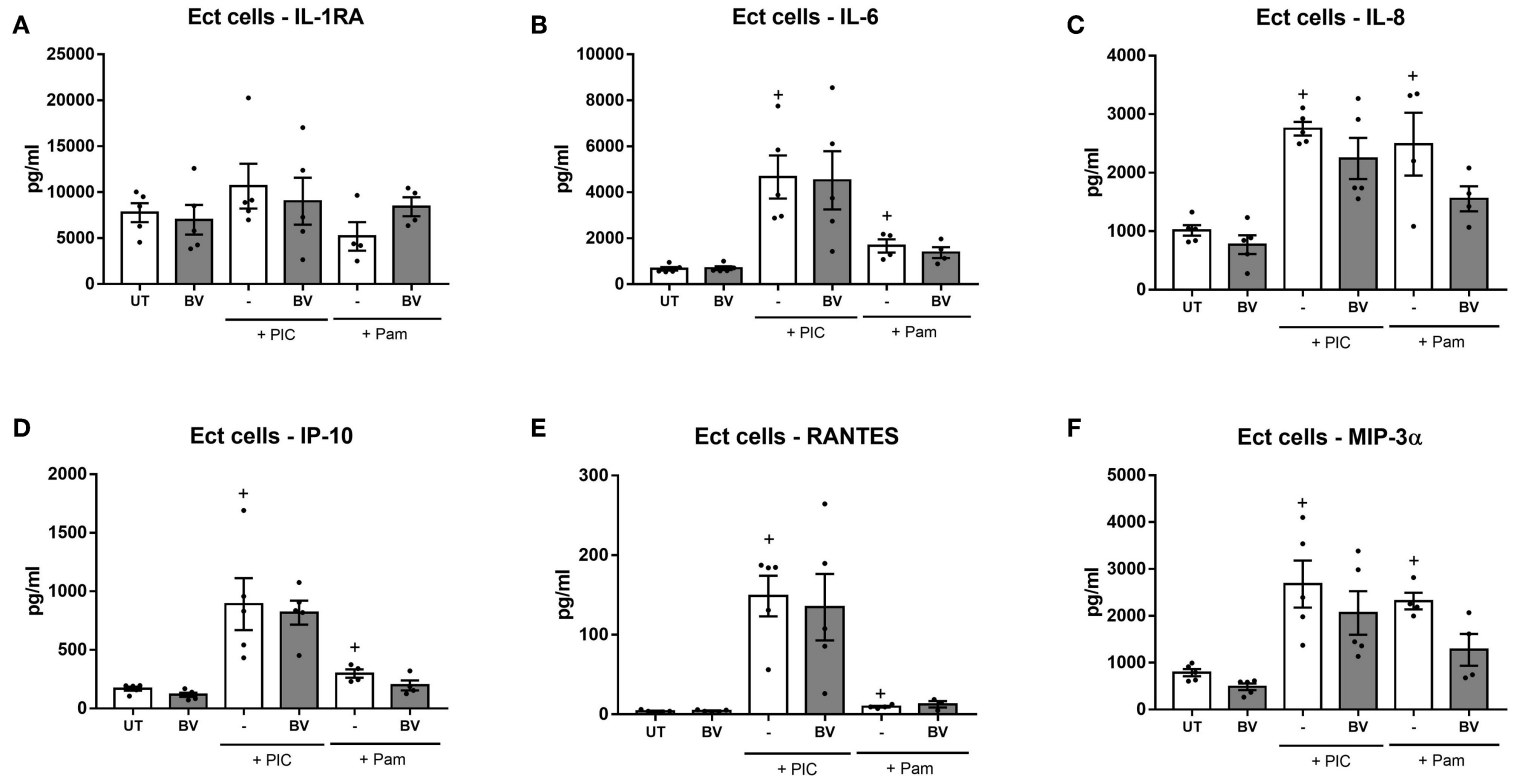
A BV vaginal microbiota metabolite combination does not alter the production of immune mediators from Ect cells treated in a transwell system. Ect cells seeded in transwells were apically treated with a mixture of vaginal microbiota metabolites at pH 7 recapitulating bacterial vaginosis (BV; 6 mM L-LA, 100 mM acetic acid, 20 mM succinic acid, 4 mM propionic acid, and 4 mM butyric acid) in the absence or presence of TLR agonists PIC (TLR3) and Pam (TLR1/2) as indicated. Cells were stimulated for 1 h and supernatant collected from the apical compartment after an additional 18 h of culture. Production of IL-1RA **(A)**, IL-6 **(B)**, IL-8 **(C)**, IP-10 **(D)**, RANTES **(E)**, and MIP-3α **(F)** was quantified using a luminex multiplex assay. Graphs show mean and standard error of the mean from *n* = 4 independent assays. Mann-Whitney tests were used to estimate statistical significance between treatment groups. + denotes a significant difference (*p* < 0.05) as compared to untreated (UT) cells. TLR, toll-like receptor; PIC, polyinosinic:polycytidylic acid; Pam, Pam3CSK4.

BV is associated with vaginal inflammation, but whether this is mediated by an effect of vaginal microbiota metabolites produced by BV-associated bacteria on the production of inflammatory mediators by epithelial cells is not known. To determine if SCFAs and succinic acid, which are found at higher concentrations in the vagina of women with BV (Al-Mushrif et al., 2000; Chaudry et al., 2004; Mirmonsef et al., 2011; Gajer et al., 2012), have immunomodulatory effects on cervicovaginal epithelial cells, a combination of vaginal microbiota metabolites representing BV conditions was established and added apically to cervicovaginal epithelial cells seeded in transwells for 1 h. This mixture contained vaginal microbial metabolites at concentrations which represented the upper limits of what is reported *in vivo* for women with BV (acetic acid 100 mM, succinic acid 20 mM, propionic acid 4 mM, and butyric acid 4 mM) and at pH 7, to reflect the higher pH found in this state ranging from pH 4.5 to 8 (Brabin et al., 2005). In contrast to the eubiotic vaginal microbiota metabolite combination, a mixture of vaginal microbiota metabolites representing BV conditions did not significantly change the production of the anti-inflammatory cytokine IL-1RA compared to untreated cells, nor did it significantly alter the production of any other immune mediators tested from either unstimulated or TLR-stimulated Ect cells (Figure 2). A similar lack of immunomodulatory effect was also observed in VK2 and primary cells (data not shown). These findings suggest that unlike L-LA under eubiotic conditions, which elicited a significant immunomodulatory effect, vaginal microbiota organic acid metabolites representative of those found in BV conditions do not alter the inflammatory response of cervicovaginal epithelial cells when added to cells for comparable periods of time.

### BV-Related Vaginal Microbiota Metabolites Have Immunomodulatory Effects on Cervicovaginal Epithelial Cells Only During Prolonged and Sustained Treatments

Given the above findings, and a previous report demonstrated a pro-inflammatory effect of SCFAs on peripheral blood cells, including neutrophils, after longer treatment periods (Mirmonsef et al., 2012b), we hypothesized that vaginal microbiota metabolites may need prolonged and sustained contact with lower female reproductive tract epithelial cells to elicit an observable effect. The transwell system allows the diffusion of molecules from the apical to basolateral compartments. Therefore, to sustain the concentration and pH of the established treatments for longer periods, cervicovaginal epithelial cells were seeded into 96-well plates. Cells were treated with the combination of vaginal microbiota metabolites representing BV conditions, with and without TLR stimulation at pH 7 for 24 h.

**Figure 3:**
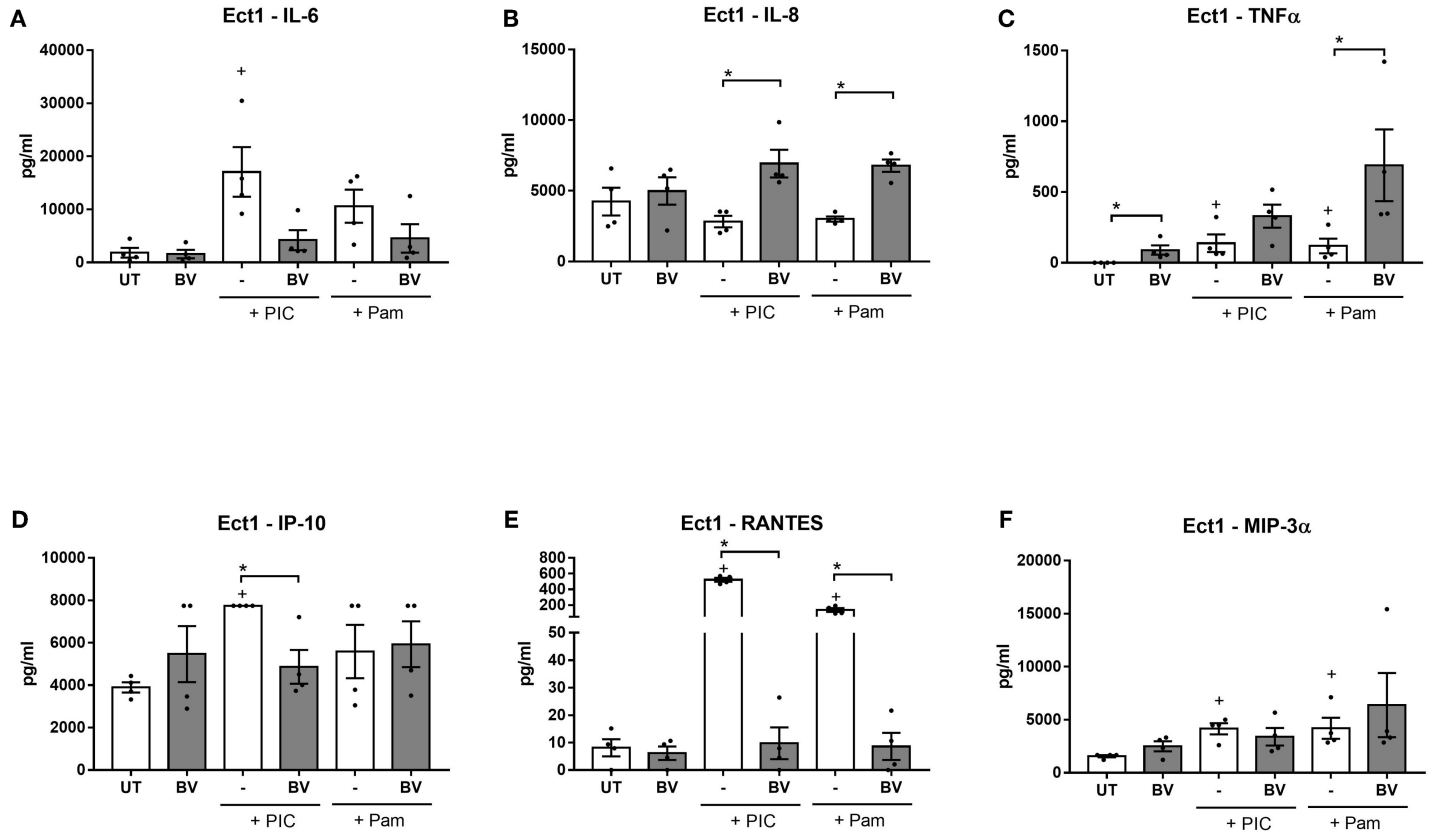
Prolonged treatment of Ect cells with BV vaginal microbiota metabolite mixture at pH 7 elicits a dysfunctional inflammatory response. Ect cells seeded in a plate format were stimulated with a mixture of vaginal microbiota metabolites recapitulating BV (6 mM L-LA, 100 mM acetic acid, 20 mM succinic acid, 4 mM propionic acid and 4 mM butyric acid) for 24 h at pH 7 in the absence or presence of stimulation with the TLR agonists PIC and Pam and production of IL-6 **(A)**, IL-8 **(B)**, TNFα **(C)**, IP-10 **(D)**, RANTES **(E)**, and MIP-3α **(F)** was quantified from supernatants. Graphs show mean and standard error of the mean from *n* = 4 independent assays. *P* ≤ 0.05 is denoted as * as determined by Mann-Whitney test. + denotes a significant difference (*p* < 0.05) as compared to untreated (UT) cells. BV, bacterial vaginosis; TLR, toll-like receptor; PIC, polyinosinic:polycytidylic acid; Pam, Pam3CSK4.

Prolonged treatments of Ect cells under these conditions with a mixture of vaginal microbiota metabolites representing BV caused an increase in the production of TNFα from unstimulated cells (*p* = 0.03, Figure 3C) and potentiated the TLR-elicited production of TNFα for Pam and BV-stimulated cells compared to cells treated with Pam alone (*p* = 0.03). A similar effect was also seen in VK2 cells (Supplementary Figure 4B). The BV vaginal microbiota metabolite mix also increased basal TNFα production from primary cervicovaginal cells, but intriguingly the opposite effect was observed in PIC-stimulated cells (Supplementary Figure 4F). The BV metabolite mixture also potentiated TLR-induced IL-8 production, with this effect being unique to Ect cells (Figure 3B and data not shown).

In contrast to this enhanced production of the pro-inflammatory mediators TNFα and IL-8, the BV vaginal microbiota metabolite mixture dampened the PIC and Pam-stimulated production of RANTES from all three cell types tested (*p* < 0.05 for all) (Figure 3E and Supplementary Figures 4D,H). Similarly, PIC-stimulated production of IP-10 was significantly inhibited by the BV vaginal microbiota metabolite mixture in all cell types (Figure 3D and Supplementary Figures 4C,G), while Pam-stimulated production was inhibited in VK2 and primary cells (*p* = 0.03 for both). Furthermore, the BV mixture inhibited basal production of IP-10, but this effect was only observed in primary cells (Supplementary Figure 4G). There was also a trend for the BV mixture to inhibit PIC-induced IL-6 production, although this was only significant in primary cells (*p* = 0.03) (Figure 3 and Supplementary Figure 4). Similar results were observed when cells were treated with a BV mixture lacking L-LA (data not shown), consistent with our previous findings that the immunomodulatory effects of L-LA are only observed at a low pH (i.e., 3.9) (Hearps et al., 2017).

Taken together, these data demonstrate that prolonged and sustained treatments of cervicovaginal epithelial cells with a mixture of SCFAs and succinic acid recreating BV conditions results in a dysregulation of inflammatory responses, with a heightened basal and TLR-simulated production of the potent inflammatory cytokine TNFα, but a dampening of TLR-elicited production of IL-6 and the chemokines RANTES and IP-10. Although most of these effects were consistent between the three cell types tested, some effects appear to be cell-type dependant (e.g., TLR-induced TNFα production in primary cells and potentiation of IL-8 production in Ect cells), highlighting the subtle but potentially important differences in the immune response of different lower female reproductive tract epithelial cell types.

### Individual Vaginal Microbiota Metabolites Elicit Dysfunctional Immunomodulatory Effects on Lower Female Reproductive Tract Epithelial Cells After Prolonged and Sustained Treatments

Having identified immunomodulatory effects of sustained treatments with a mixture of BV vaginal microbiota metabolites, we sought to identify which individual metabolite might be responsible for these dysfunctional effects. Lower female reproductive tract epithelial cells were therefore treated with individual SCFAs or succinic acid in 96 well plates under the same concentrations and pH conditions as above.

**Figure 4:**
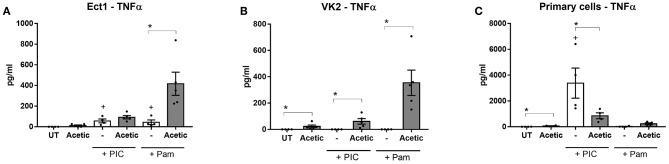
Acetic acid alone increases basal and TLR-elicited TNF production from lower female reproductive tract epithelial cells. Ect **(A)**, VK2 **(B)**, and primary cervicovaginal epithelial cells **(C)** seeded into plates were treated with 100 mM acetic acid (Acetic) at pH 7 for 24 h in the absence or presence of stimulation with the TLR agonists PIC and Pam as indicated and production of TNFα was quantified from supernatants. Graphs show mean and standard error of the mean from *n* = 4 independent assays. *P* ≤ 0.05 are denoted as * as determined by Mann-Whitney test. + denotes a significant difference (*p* < 0.05) as compared to untreated (UT) cells. TLR, toll-like receptor; PIC, polyinosinic:polycytidylic acid; Pam, Pam3CSK4.

The pro-inflammatory effect of the BV mixture on increased TNFα production was largely recapitulated by treatment of cells with acetic acid alone (Figures 4A–C) with a consistent effect observed for all three cell types. Treatment with acetic acid alone also reproduced the potentiation of TLR-induced TNFα production in Ect and VK2 cells, and the converse effect in primary epithelial cells (Figures 4A–C). Butyric acid alone had a similar but non-significant effect on enhancing TLR-elicited TNFα production in Ect cells (Supplementary Figure 5), while no effects were observed for succinic acid or propionic acid alone in any cell type tested (data not shown). These data suggest that acetic acid, and to a lesser extent butyric acid, are likely the metabolites responsible for the pro-inflammatory effects of the BV mixture on TNFα production in these cells.

To identify the vaginal microbiota metabolites responsible for the potentiation of TLR-induced IL-8 production observed in Ect cells, we analyzed the effect of acetic, succinic, propionic, and butyric acids individually but were unable to identify any single metabolite which recapitulated this effect (data not shown). These data suggest the effects may be due to the combination of these metabolites. Interestingly, butyric acid alone showed a potentiation of TLR-induced IL-8 production in VK2 cells (Supplementary Figure 6A), which was not seen in Ect or primary cells, reinforcing the differences that exist between these cell types.

**Figure 5:**
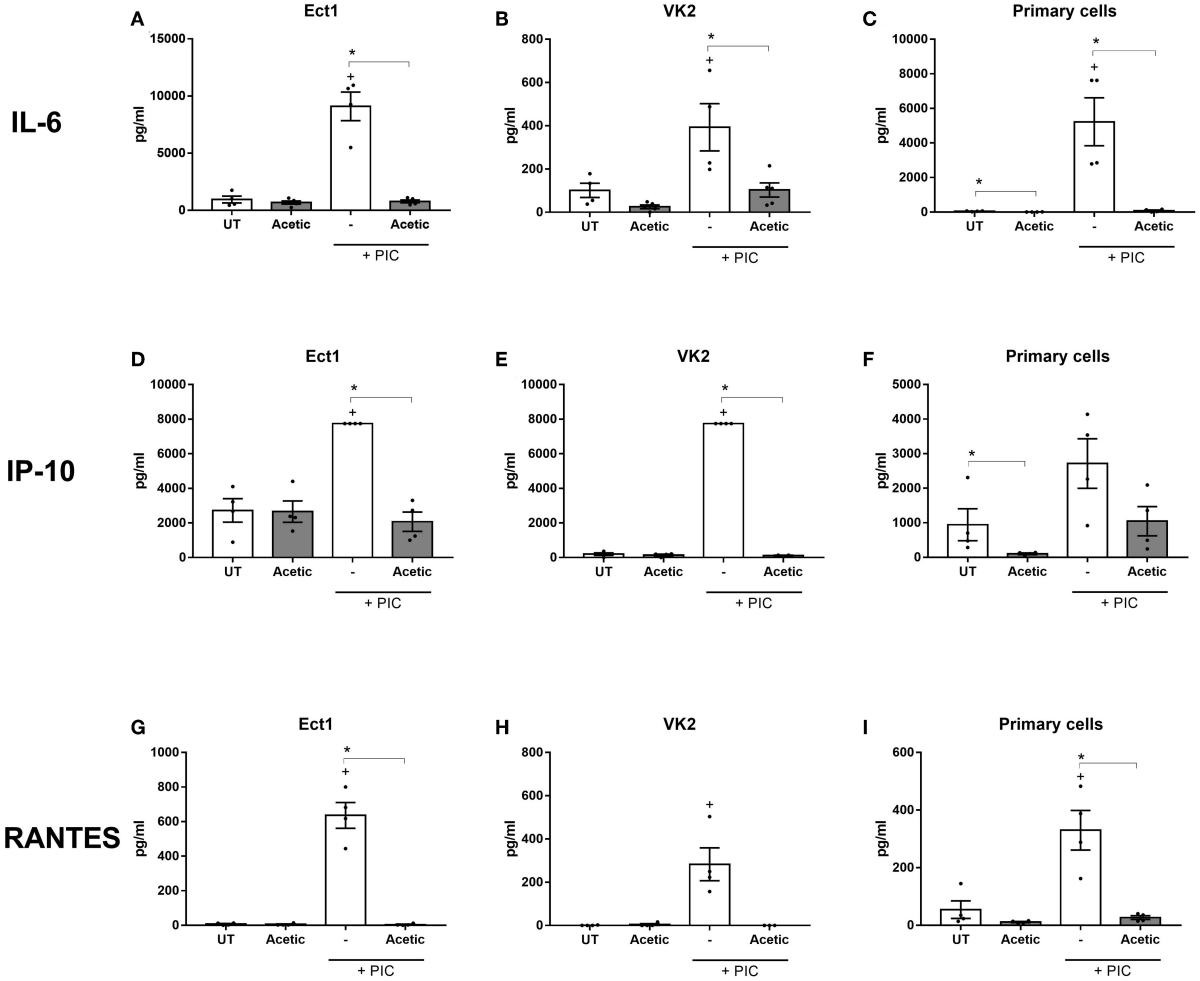
Acetic acid alone inhibits TLR-elicited IL-6, IP-10, and RANTES production from lower female reproductive tract epithelial cells. Ect (left panels), VK2 (center panels), and primary cervicovaginal epithelial cells (right panels) seeded into plates were treated with 100 mM acetic acid (Acetic) at pH 7 for 24 h in the absence or presence of stimulation with the TLR agonist PIC. Production of IL-6 **(A–C)**, IP-10 **(D–F)**, and RANTES **(G–I)** was quantified from supernatants. Graphs show mean and standard error of the mean from *n* = 4 independent assays. *P* ≤ 0.05 are denoted as * (as determined by Mann-Whitney test). + denotes a significant difference (*p* < 0.05) as compared to untreated (UT) cells. PIC, polyinosinic:polycytidylic acid.

When we interrogated the vaginal microbiota metabolites driving the dampening effect of the BV mixture on TLR-induced chemokine production we also found evidence implicating acetic acid. Treatment with acetic acid alone resulted in a striking inhibition of TLR3-induced RANTES, IP-10, and IL-6 production which was consistent across Ect, VK2 and primary epithelial cells (Figures 5A–I), suggesting this metabolite is contributing significantly to the immune dampening effect observed with the BV vaginal microbiota mixture. Butyric acid alone was also able to recapitulate the dampening effects on TLR-induced RANTES and IP-10 production, but primarily in VK2 cells (Supplementary Figure 6), and not in Ect cells (data not shown). Succinic acid also trended toward impairing TLR-elicited IL-6 production in Ect and VK2 cells, while no significant immunomodulatory effects were observed with propionic acid alone (data not shown). These experiments, which aimed to deconstruct the immune effects of a vaginal microbiota metabolite mixture reflecting a state of BV, identified acetic acid as a significant metabolite capable of eliciting seemingly conflicting immunomodulatory effects on a range of lower female reproductive tract epithelial cell targets. Our findings also suggest that butyric acid may be a potent immune modulating factor primarily for VK2 cells, indicating that different vaginal microbiota metabolites can have cell- and context- specific roles on modulating inflammation in the lower female reproductive tract.

## Discussion

A vaginal microbiota dominated by beneficial *Lactobacillus* spp. is associated with protection against viral and bacterial pathogens (Conti et al., 2009; Graver and Wade, 2011; O'Hanlon et al., 2011; Aldunate et al., 2013; Gong et al., 2014; Gosmann et al., 2017). Conversely, several studies have demonstrated a relationship between a non-optimal vaginal microbiota such as in BV and genital inflammation, and an increased risk of acquiring HIV (Sewankambo et al., 1997; Taha et al., 1998; Atashili et al., 2008; Masson et al., 2015; Gosmann et al., 2017). However, the vaginal microbiota produces a range of metabolites, peptides and proteins (Shaw et al., 2007; Cruciani et al., 2013; Vitali et al., 2015) and the discrete factors responsible for the aforementioned effects are not completely known, nor is it understood how these factors may interact with each other within the complex female reproductive tract milieu.

Here and previously, we have demonstrated a potent ability of L-LA, a major metabolite produced by lactobacilli, to elicit an anti-inflammatory effect as well as inhibit pro-inflammatory cytokine and chemokine production from cervicovaginal epithelial cells (Hearps et al., 2017). We describe for the first time the effect of L-LA on dampening TLR-elicited IP-10 production which is of special interest given the association of this cytokine with increased HIV transmission in women (Masson et al., 2015). In this study, we confirmed the anti-inflammatory activity of LA is maintained in the presence of an organic acid metabolite mixture relevant to vaginal eubiosis. This immunomodulatory effect was demonstrated by an increased production of the anti-inflammatory cytokine IL-1RA, which antagonizes the IL-1 receptor and inhibits pro-inflammatory signals from the IL-1 cytokines (Arend, 2002), and by a small decrease in the production of the pro-inflammatory factors IL-6 and MIP-3α. Moreover, in TLR stimulated cervicovaginal epithelial cells a eubiotic vaginal microbiota metabolite mixture containing L-LA dampened the production of pro-inflammatory factors associated with vaginal inflammation and recruitment of HIV target cells to the female reproductive tract epithelium (Sturm-Ramirez et al., 2000; Narimatsu et al., 2005; de Jong et al., 2008; Masson et al., 2015; Arnold et al., 2016; Gosmann et al., 2017).

Cervicovaginal epithelial cell secretion of MIP-3α has been shown to attract Langerhans cells (Cremel et al., 2005; Berlier et al., 2006), which although controversial, have been proposed to act as a “Trojan horse” facilitating HIV dissemination to lymph nodes (Cavrois et al., 2008; Matsuzawa et al., 2017). In this regard, the effect of L-LA on dampening MIP-3α production may also be important for inhibiting HIV transmission. The ability of L-LA to inhibit pro-inflammatory factors associated with HIV transmission, even when present within a complex vaginal microbiota metabolite mix, suggests this metabolite may be an important driver of the protective effect of lactobacillus-dominated vaginal microbiota against HIV transmission observed *in vivo*. These L-LA-elicited effects also support the potential use of protonated LA administered vaginally, or vaginal probiotic treatment using lactobacilli to sustain the production of LA, to maintain an anti-inflammatory state *in vivo* and help protect against HIV infection.

In contrast to the striking immunomodulatory effects observed after a brief 1 h incubation of epithelial cells with L-LA and L-LA-containing metabolite mixtures, stimulation of cells for a similar time with a combination of vaginal microbiota metabolites representing BV conditions had no such effect. We also assessed the effect of individual SCFA and succinic acid, at the higher concentrations and pH reflective of BV, on cervicovaginal epithelial cells and similarly found no significant effects, even when metabolites were added at a lower pH of 5.5 (data not shown). This highlights the unique and potent effect of L-LA, and lends further weight to its potential therapeutic use to promote optimal female reproductive tract health. We did however find that sustained and prolonged treatments of epithelial cells with BV-relevant metabolites elicited somewhat conflicting and potentially dysfunctional changes in the inflammatory response of these cells. Consistent with previous findings demonstrating a pro-inflammatory effect of BV-relevant SCFA on peripheral blood mononuclear cells and neutrophils (Mirmonsef et al., 2012b), we found a combination of vaginal microbiota metabolites representing BV increased basal and TLR-induced production of TNFα, which was largely consistent across all cell types tested. Further analyses suggested this effect was due at least in part to the action of acetic acid. However, this pro-inflammatory effect was contrasted by a significant impairment of TLR-induced production of other inflammatory factors, which again appeared to be mediated by acetic acid, and to a lesser extent butyric acid. These findings indicate that sustained treatment with BV-relevant vaginal microbiota metabolites dysregulates the immune response of cervicovaginal epithelial cells, with the net effect of these anti-and pro-inflammatory responses remaining unclear. However, it is worth noting that a similar dichotomous pattern in the levels of pro-inflammatory mediators was observed in the cervicovaginal fluid of HIV-uninfected women in South Africa with BV (Masson et al., 2014). In this study elevated levels of the pro-inflammatory cytokines IL-1α, IL-1β and TNFα but decreased levels of several pro-inflammatory chemokines including IP-10 and MIP-α was reported (Masson et al., 2014). While the *in vivo* cervicovaginal milieu is more complex than our *in vitro* analysis of BV-associated SCFAs on cervicovaginal epithelial cells, it is possible that the elevated levels of TNFα and decreased levels of IP-10 may in part be mediated by the action of SCFAs, and in particular acetic acid, on the cervicovaginal mucosa.

These experiments also identified cell-specific immunomodulatory effects, with the metabolite butyric acid appearing to have an immunomodulatory effect in VK2 but not Ect or primary epithelial cells, while acetic acid showed a largely consistent effect on all cell types. This is consistent with other studies reporting a cell-type dependent response to SCFAs (Park et al., 2007; Vinolo et al., 2011; Mirmonsef et al., 2012b; Kim et al., 2013) and highlights the importance of using physiologically relevant cell types in studies of vaginal microbiota metabolites. Our data also suggest there may be differences in the epithelial microenvironment at various sites within the female reproductive tract due to variations in the response of resident epithelial cells to microbiota products. Similar site-specific variations have been shown regarding the effect of the antiretroviral drug tenofovir, which increases pro-inflammatory cytokine production from endometrial and ectocervical, but not endocervical epithelial cells (Biswas et al., 2014). Another intriguing observation, which was unique to primary cells, was the conflicting effect of the BV metabolite mixture on TNFα production, in that it enhanced basal TNFα production but inhibited PIC-induced TNFα production. This effect obviously requires further investigation in different types of primary cells, but alludes to the fact that unlike LA, which exhibits a more consistent and universal anti-inflammatory effect on basal and stimulated cells, vaginal microbiota metabolites present in BV may have a more variable and nuanced effect which is influenced by cell and pathogen-specific factors.

The inflammatory state present in BV may be driven by vaginal microbiota metabolites other than those assessed here, or by other vaginal microbial products. A number of studies have profiled the BV metabolome and proteome and identified substantial metabolic and proteomic changes in vaginal fluid from BV-affected women (Srinivasan et al., 2015; Arnold et al., 2016; Borgdorff et al., 2016) with one study identifying 17 previously unreported molecules enriched in BV samples (Vitali et al., 2015). The impact of these factors on vaginal inflammation remains to be determined. However, the cause of female reproductive tract inflammation may not only be due to the presence of pro-inflammatory BV-related organisms (and the effect of their metabolites), but also the additional effect of an absence of anti-inflammatory factors such as LA.

A limitation of this study was that it analyzed the effect of vaginal microbiota metabolites on cervicovaginal epithelial cell monolayers. The cervicovaginal epithelium *in vivo* is multilayered and contains various cell types including immune cells. Thus, it will be important to confirm the effects of eubiotic and BV metabolites in a more physiologically relevant system, such as vaginal tissue, or the 3D EpiVaginal^TM^ tissue model (MatTek, Ashland, MA) we have previously used to validate the anti-inflammatory effects of LA (Hearps et al., 2017). Furthermore, we assessed concentrations of vaginal microbiota metabolites reflective of those found in the vaginal lumen; cells present deeper within the vaginal epithelium may be exposed to different, and likely lower, concentrations of metabolites than those analyzed here. The effects of eubiotic metabolite mixtures were assessed here at pH 3.9, and although we did not find a significant immunomodulatory effect of pH 3.9 media alone on cervicovaginal cells in a previous study (Hearps et al., 2017), acidity has been shown to dampen inflammatory responses of other types of epithelial cells (Hackett et al., 2016). It therefore remains possible that low pH may be contributing to the anti-inflammatory properties of the eubiotic metabolites shown here.

In this study we focused on L-LA due to our previous findings regarding its superior virucidal activity against HIV (Aldunate et al., 2013), and a similar anti-inflammatory effect of both L- and D-LA (Hearps et al., 2017), but it would be of interest to confirm the current findings with D-LA and/or various ratios of L- and D-LA reflective of those found in women colonized with different lactobacilli. We assessed responses to TLR3 and TLR1/2 agonists here given our previous findings of similar anti-inflammatory effects of LA irrespective of TLR agonist used (Hearps et al., 2017), thus potential differences regarding effects of the vaginal microbial metabolites studied here on responses to other TLR agonists remain to be confirmed. Although this study investigated five predominant organic acid metabolites detected in women with optimal and non-optimal microbiota, other metabolites including pyruvic and isobutyric acid are detected in the female genital tract and may also have immunomodulatory effects (Stanek et al., 1992). Notwithstanding these limitations, this study advances our knowledge on the effects of single vaginal microbiota metabolites that comprise the complex cervicovaginal environment and support our previous findings showing the beneficial effects of LA in preventing inflammation known to be associated with increased risk of HIV acquisition. Future *in vivo* studies assessing the efficacy of topically applied LA in maintaining a eubiotic vaginal state and preventing BV and associated genital inflammation will be important for informing new prevention strategies to mitigate transmission of HIV and other sexually transmitted infections.

## Data Availability Statement

The datasets generated for this study are available on request to the corresponding author.

## Author Contributions

GT and AH conceived the study. DD-D and DT generated experimental data. AH, DD-D, JH, RG, and GT provided input into study design. DD-D performed data analysis and wrote the manuscript with assistance from AH and GT. All authors reviewed the final version of the manuscript.

### Conflict of Interest

GT and AH are coinventors on patent application AU201501042 and United States Patent No. US 9,801,839 B2 claiming the anti-inflammatory effects of lactic acid. The remaining authors declare that the research was conducted in the absence of any commercial or financial relationships that could be construed as a potential conflict of interest.
