# Supplementary material for: Distinct Immune Responses Elicited From Cervicovaginal Epithelial Cells by Lactic Acid and Short Chain Fatty Acids Associated With Optimal and Non-optimal Vaginal Microbiota
Source: Front Cell Infect Microbiol. 2020 Jan 10;9:446. doi: 10.3389/fcimb.2019.00446 (PMC6965070; doi:10.3389/fcimb.2019.00446)
Supplement: Supplementary file 1 [file Presentation_1.PPTX]

## Slide 1
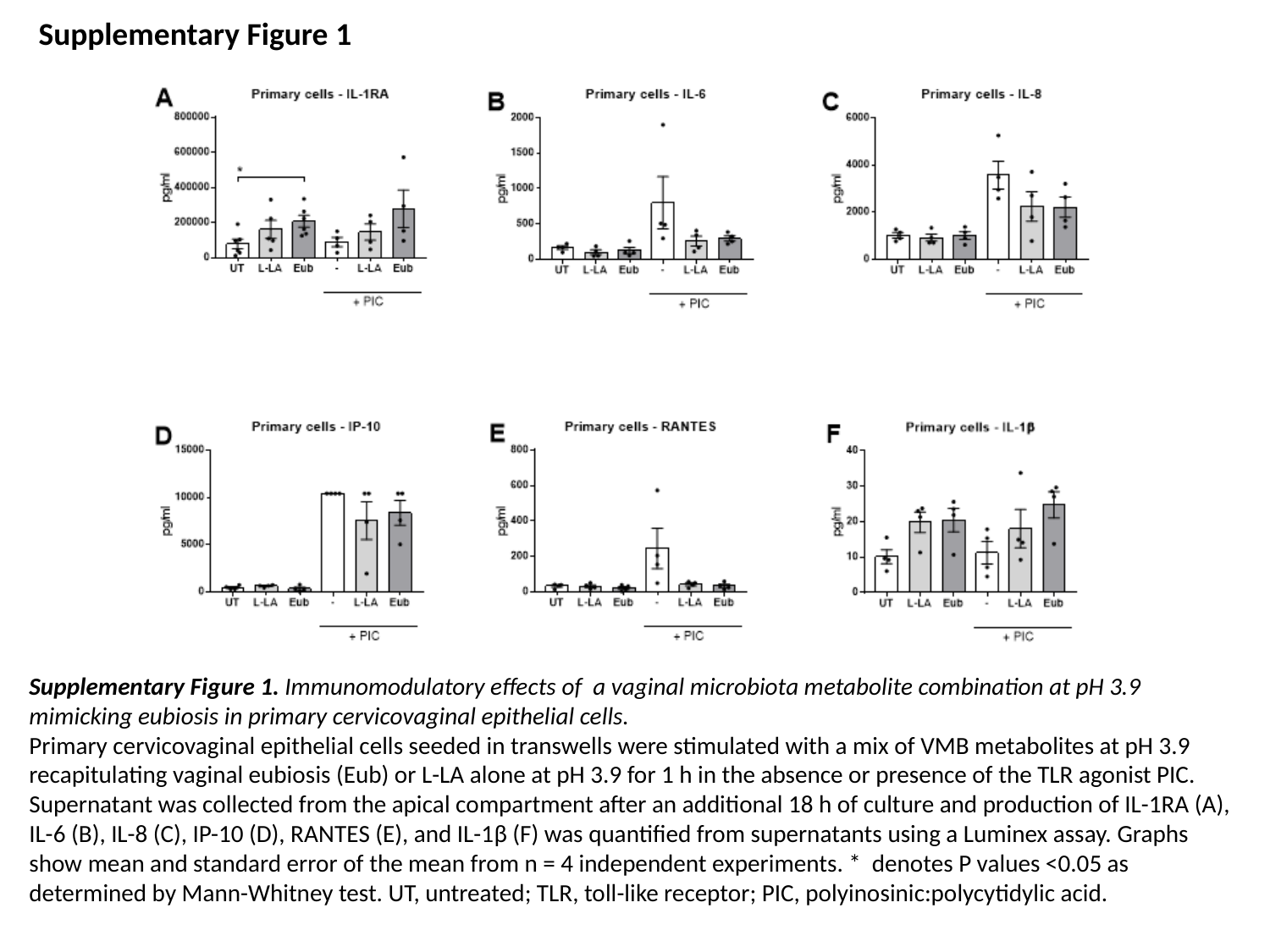

Supplementary Figure 1
Supplementary Figure 1. Immunomodulatory effects of a vaginal microbiota metabolite combination at pH 3.9 mimicking eubiosis in primary cervicovaginal epithelial cells.
Primary cervicovaginal epithelial cells seeded in transwells were stimulated with a mix of VMB metabolites at pH 3.9 recapitulating vaginal eubiosis (Eub) or L-LA alone at pH 3.9 for 1 h in the absence or presence of the TLR agonist PIC. Supernatant was collected from the apical compartment after an additional 18 h of culture and production of IL-1RA (A), IL-6 (B), IL-8 (C), IP-10 (D), RANTES (E), and IL-1β (F) was quantified from supernatants using a Luminex assay. Graphs show mean and standard error of the mean from n = 4 independent experiments. * denotes P values <0.05 as determined by Mann-Whitney test. UT, untreated; TLR, toll-like receptor; PIC, polyinosinic:polycytidylic acid.

## Slide 2
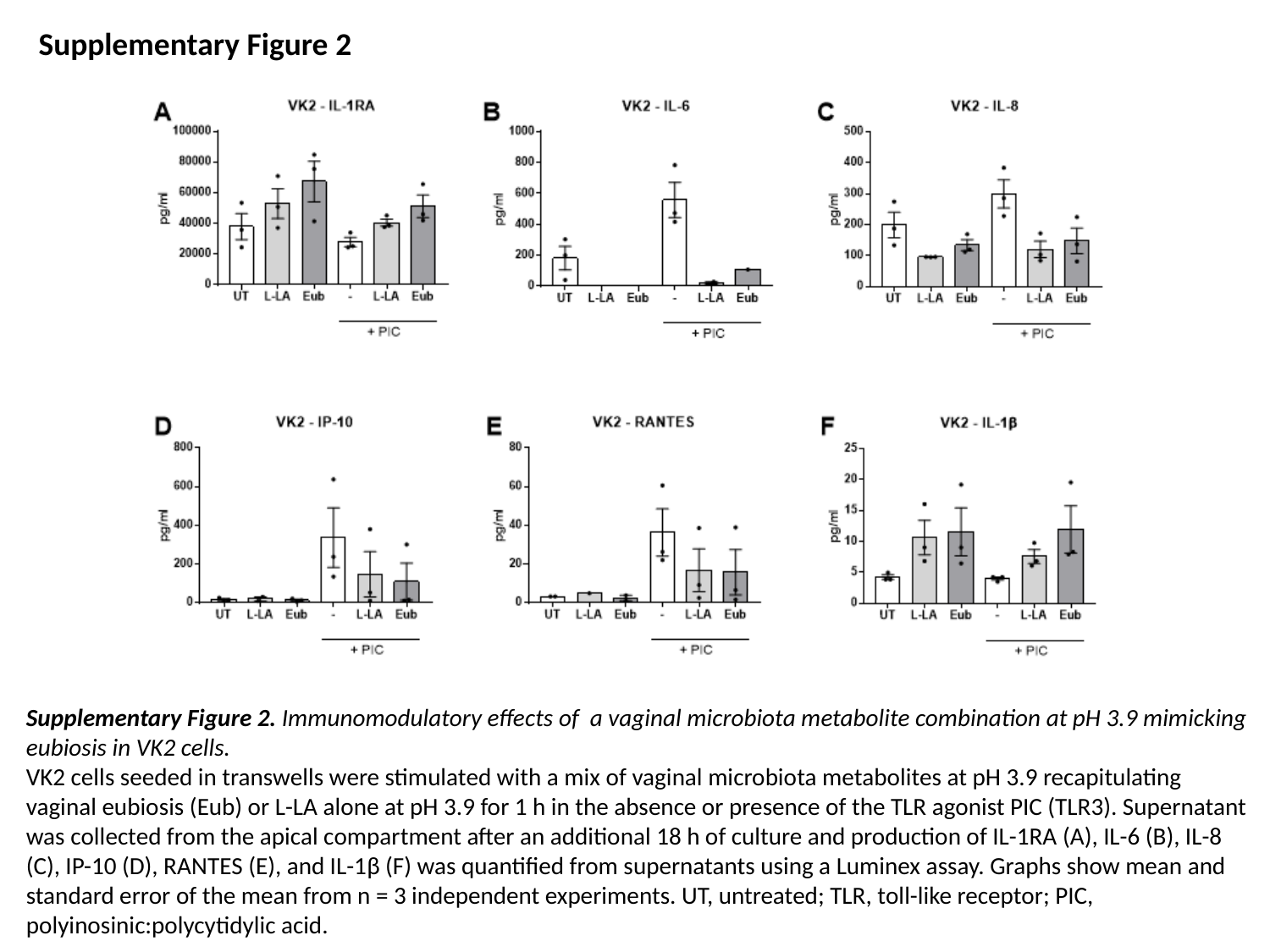

Supplementary Figure 2
Supplementary Figure 2. Immunomodulatory effects of a vaginal microbiota metabolite combination at pH 3.9 mimicking eubiosis in VK2 cells.
VK2 cells seeded in transwells were stimulated with a mix of vaginal microbiota metabolites at pH 3.9 recapitulating vaginal eubiosis (Eub) or L-LA alone at pH 3.9 for 1 h in the absence or presence of the TLR agonist PIC (TLR3). Supernatant was collected from the apical compartment after an additional 18 h of culture and production of IL-1RA (A), IL-6 (B), IL-8 (C), IP-10 (D), RANTES (E), and IL-1β (F) was quantified from supernatants using a Luminex assay. Graphs show mean and standard error of the mean from n = 3 independent experiments. UT, untreated; TLR, toll-like receptor; PIC, polyinosinic:polycytidylic acid.

## Slide 3
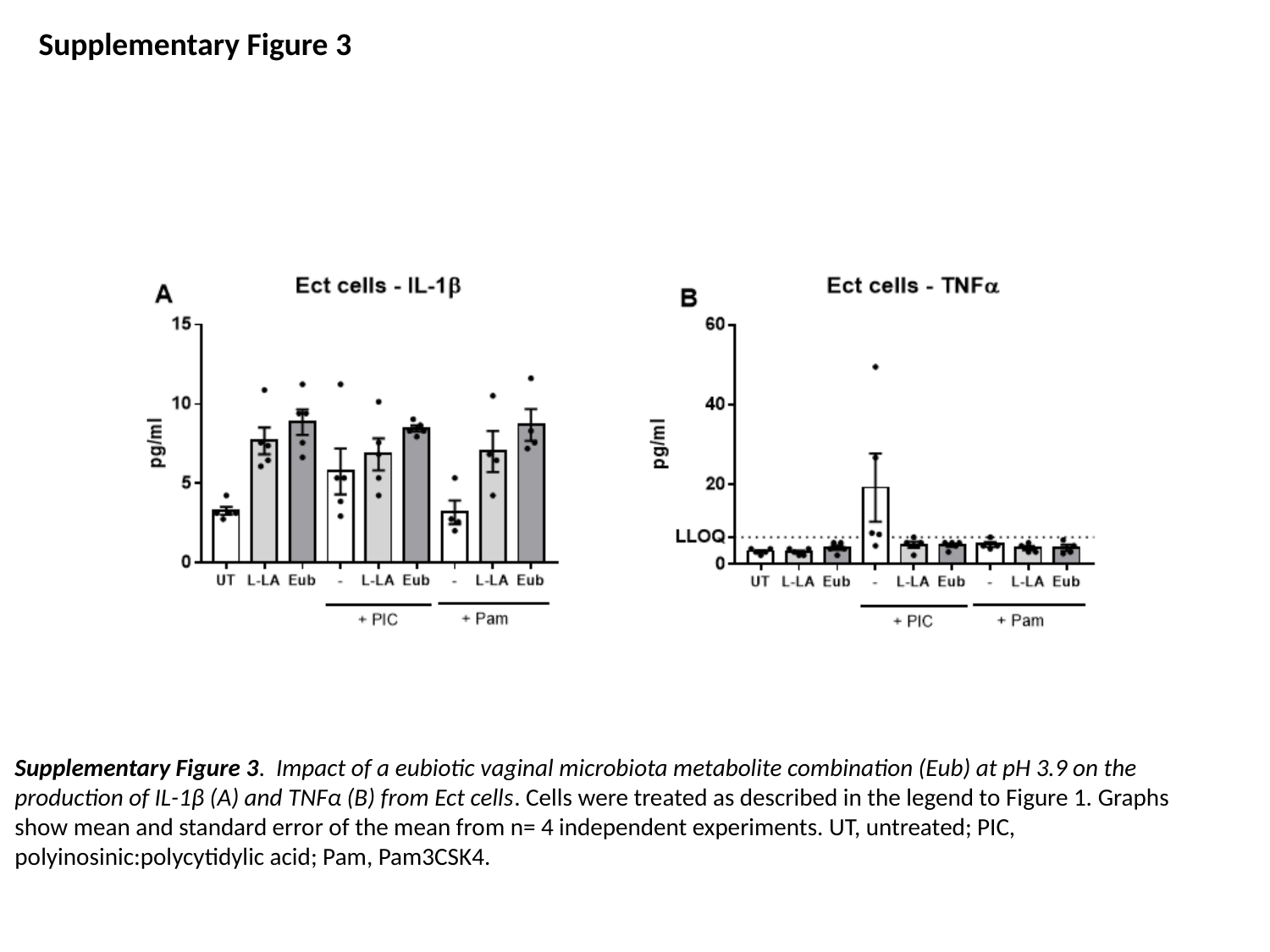

Supplementary Figure 3
Supplementary Figure 3. Impact of a eubiotic vaginal microbiota metabolite combination (Eub) at pH 3.9 on the production of IL-1β (A) and TNFα (B) from Ect cells. Cells were treated as described in the legend to Figure 1. Graphs show mean and standard error of the mean from n= 4 independent experiments. UT, untreated; PIC, polyinosinic:polycytidylic acid; Pam, Pam3CSK4.

## Slide 4
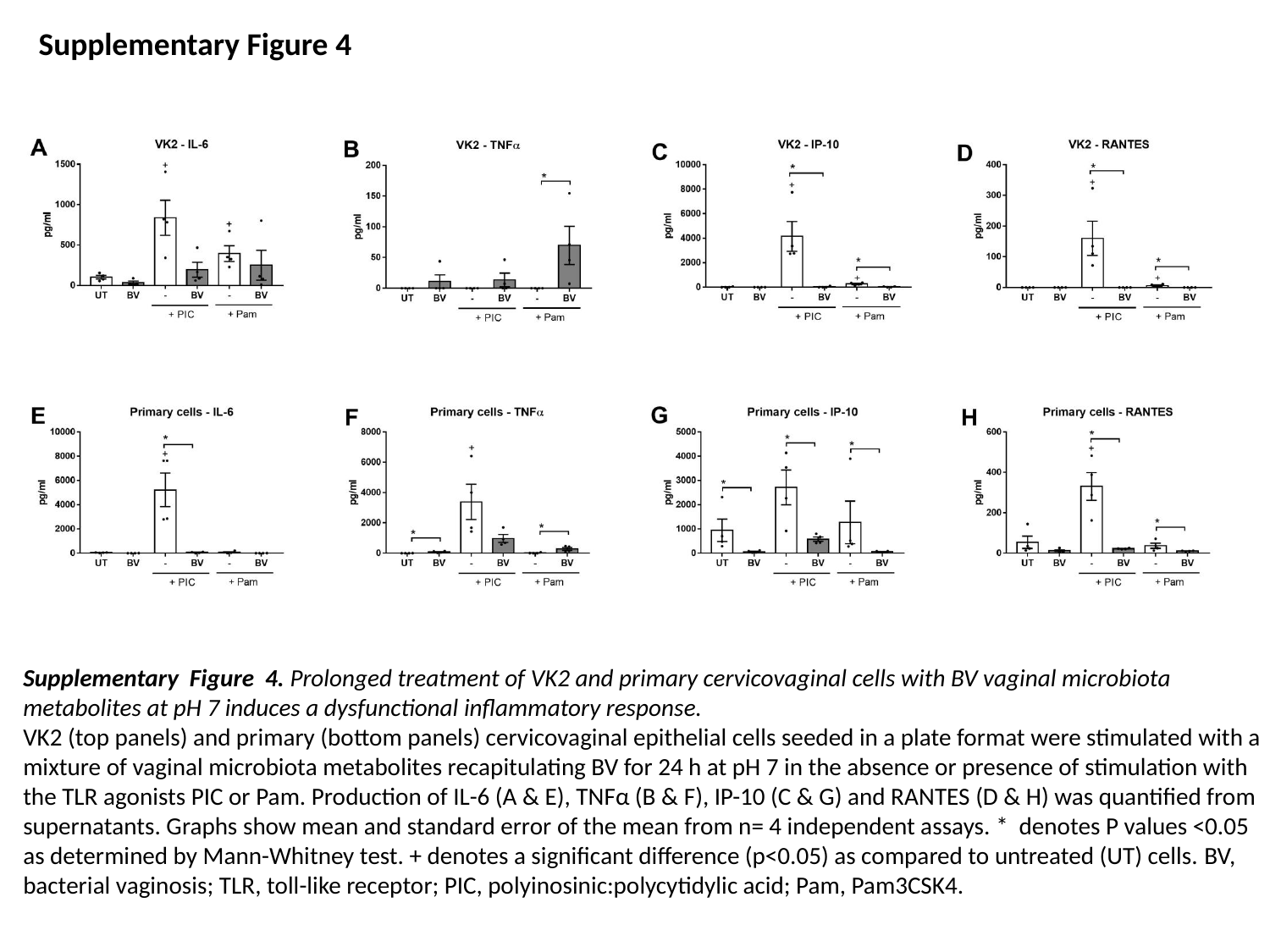

Supplementary Figure 4
Supplementary Figure 4. Prolonged treatment of VK2 and primary cervicovaginal cells with BV vaginal microbiota metabolites at pH 7 induces a dysfunctional inflammatory response.
VK2 (top panels) and primary (bottom panels) cervicovaginal epithelial cells seeded in a plate format were stimulated with a mixture of vaginal microbiota metabolites recapitulating BV for 24 h at pH 7 in the absence or presence of stimulation with the TLR agonists PIC or Pam. Production of IL-6 (A & E), TNFα (B & F), IP-10 (C & G) and RANTES (D & H) was quantified from supernatants. Graphs show mean and standard error of the mean from n= 4 independent assays. * denotes P values <0.05 as determined by Mann-Whitney test. + denotes a significant difference (p<0.05) as compared to untreated (UT) cells. BV, bacterial vaginosis; TLR, toll-like receptor; PIC, polyinosinic:polycytidylic acid; Pam, Pam3CSK4.

## Slide 5
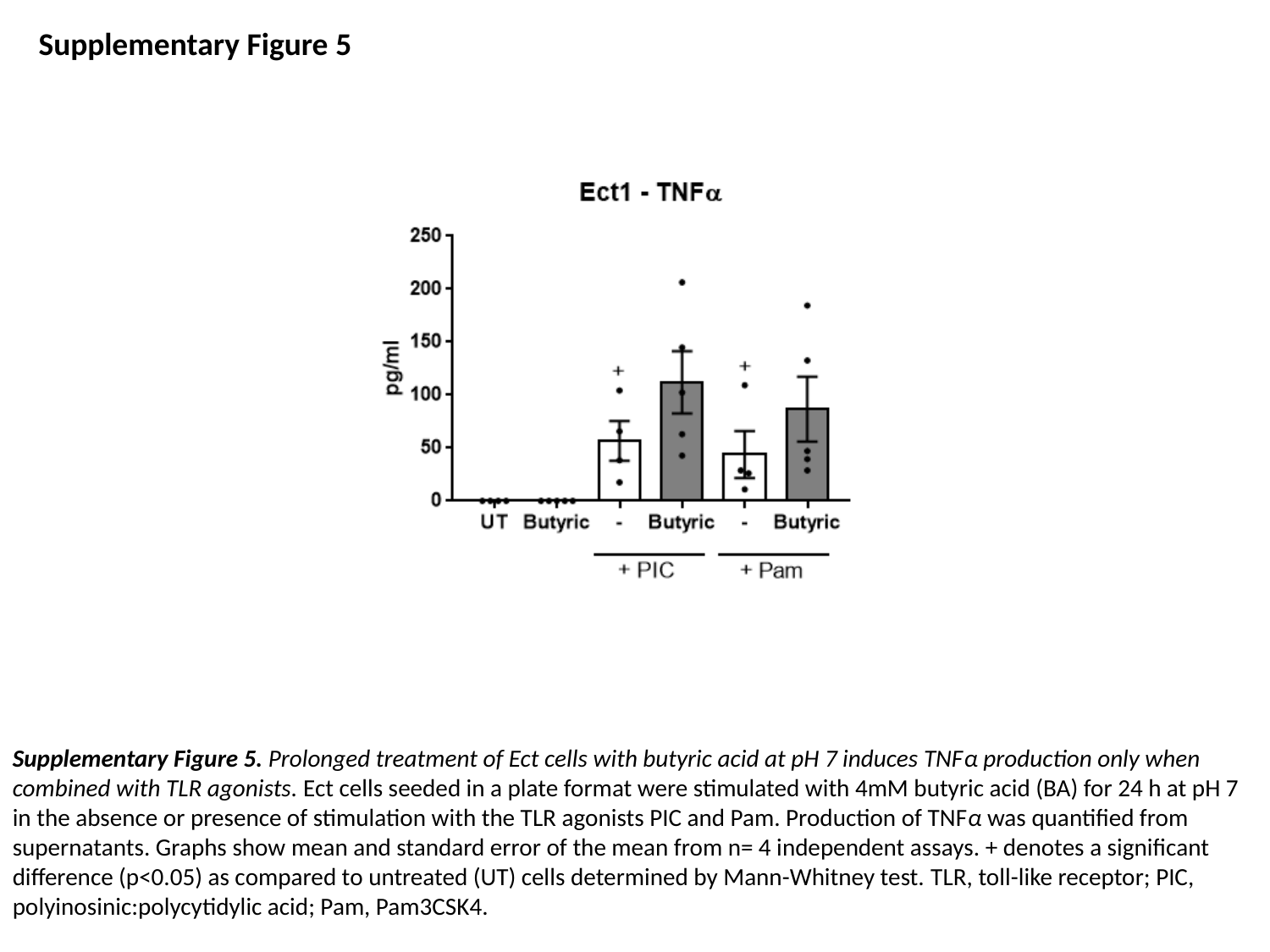

Supplementary Figure 5
Supplementary Figure 5. Prolonged treatment of Ect cells with butyric acid at pH 7 induces TNFα production only when combined with TLR agonists. Ect cells seeded in a plate format were stimulated with 4mM butyric acid (BA) for 24 h at pH 7 in the absence or presence of stimulation with the TLR agonists PIC and Pam. Production of TNFα was quantified from supernatants. Graphs show mean and standard error of the mean from n= 4 independent assays. + denotes a significant difference (p<0.05) as compared to untreated (UT) cells determined by Mann-Whitney test. TLR, toll-like receptor; PIC, polyinosinic:polycytidylic acid; Pam, Pam3CSK4.

## Slide 6
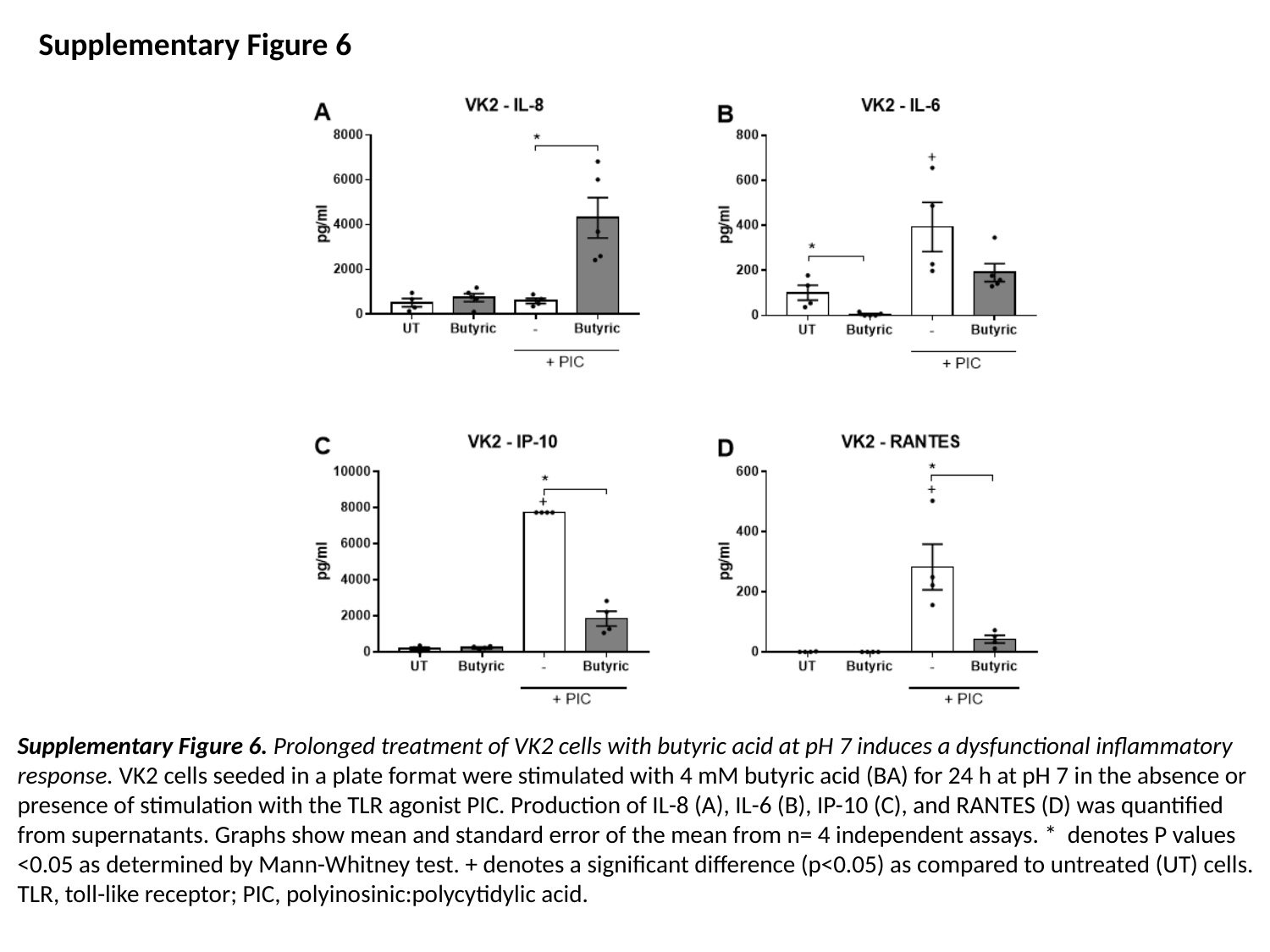

Supplementary Figure 6
Supplementary Figure 6. Prolonged treatment of VK2 cells with butyric acid at pH 7 induces a dysfunctional inflammatory response. VK2 cells seeded in a plate format were stimulated with 4 mM butyric acid (BA) for 24 h at pH 7 in the absence or presence of stimulation with the TLR agonist PIC. Production of IL-8 (A), IL-6 (B), IP-10 (C), and RANTES (D) was quantified from supernatants. Graphs show mean and standard error of the mean from n= 4 independent assays. * denotes P values <0.05 as determined by Mann-Whitney test. + denotes a significant difference (p<0.05) as compared to untreated (UT) cells. TLR, toll-like receptor; PIC, polyinosinic:polycytidylic acid.
